# Differential expression of CD64 in patients with *Mycobacterium tuberculosis* infection: A potential biomarker for clinical diagnosis and prognosis

**DOI:** 10.1111/jcmm.16004

**Published:** 2020-11-08

**Authors:** Qianqian Liu, Yan Gao, Qinfang Ou, Yuzhen Xu, Zhe Zhou, Ting Li, Yi Lu, Feng Sun, Xian Zhou, Yang Li, Lingyun Shao, Wenhong Zhang

**Affiliations:** ^1^ Department of Infectious Diseases Huashan Hospital Fudan University Shanghai China; ^2^ Department of Pulmonary Diseases Wuxi Infectious Diseases Hospital Wuxi China; ^3^ BD Medical Devices (Shanghai) Co., Ltd Shanghai China; ^4^ Shanghai Qianghan Medical Devices Co., Ltd Shanghai China; ^5^ Key Laboratory of Medical Molecular Virology (MOE/MOH) Institutes of Biomedical Science Shanghai Medical College Fudan University Shanghai China; ^6^ State Key Laboratory of Genetic Engineering School of Life Science Fudan University Shanghai China; ^7^ National Clinical Research Center for Aging and Medicine Huashan Hospital Fudan University Shanghai China

**Keywords:** CD64, diagnosis, neutrophil, prognosis, tuberculosis

## Abstract

To evaluate the clinical utility of neutrophil (n)CD64 index to diagnose pulmonary tuberculosis (PTB) and extrapulmonary TB (ePTB) and to predict the outcome of *Mycobacterium tuberculosis* infection. We recruited 189 patients with active TB and 140 controls and measured the differential expression of nCD64 index using flow cytometry. The receiver operating characteristics (ROC) curve analysis was performed to estimate the diagnostic performance of the nCD64 index and T‐SPOT.TB assay for the diagnosis of TB. Furthermore, we analysed whether the nCD64 index in patients with TB was correlated with inflammatory indicators. Finally, we assessed the prognosis of patients by following the dynamic changes of the nCD64 index once a week. The nCD64 index was significantly higher in active TB group (PTB and ePTB), than in the anti‐TB and healthy controls (HC) groups. The sensitivity and specificity of nCD64 index for the differential diagnosis of PTB and pneumonia (PN) patients were 68.33% and 77.55%, respectively. The sensitivity and specificity of nCD64 index for the diagnosis of tuberculous meningitis (TBM) were 53.85% and 100%, respectively. Furthermore, there was a weak correlation between the nCD64 index and inflammatory indicators. More importantly, with the improvement in patient condition, the nCD64 index started to decline in the first week of anti‐TB therapy and significantly decreased at 4 weeks after treatment. Our study demonstrated that the CD64 assay is a rapid, non‐invasive and stable method for clinical application, and the nCD64 index can serve as a potential biomarker for the diagnosis and prognosis of TB.

## 1 INTRODUCTION

Tuberculosis (TB), caused by *Mycobacterium tuberculosis* infection, is a major global health concern. There is a lack of specific biomarkers for the diagnosis and prognosis of patients with active TB, especially in patients with extrapulmonary TB (ePTB), whose lesions occur in the meninges, pleura, lymph nodes or bones, except the lungs. Hence, there is a need for novel methods or markers to diagnose pulmonary TB (PTB) and ePTB and to predict the outcome of *M. tuberculosis* infection.

CD64, one of the immunoglobulin (Ig)G Fc receptors (Fcγ RI), is constitutively expressed on monocytes and macrophages but hardly expressed on lymphocytes.^1^ Under normal conditions, CD64 is expressed on resting neutrophils at a negligible level, but it increases by 10‐fold at 4‐6 hours after inflammatory response or stimulation by proinflammatory cytokines, such as granulocyte colony‐stimulating factor (G‐CSF) and interferon (IFN)‐γ, and thus plays an important role in the immune response to infection.^2^, ^3^, ^4^ Several studies have reported that neutrophil CD64 (nCD64) can be used as an early diagnostic biomarker in patients with sepsis, especially bacterial infection.^5^, ^6^, ^7^, ^8^, ^9^, ^10^, ^11^ However, only a few studies have evaluated the diagnostic and prognostic utility of nCD64 in patients with *M. tuberculosis* infection, especially in patients with ePTB.

Therefore, our study was designed to address the above concerns. In the present study, we first analysed the differential expression of nCD64 in patients with PTB, ePTB, long‐term anti‐TB treatment (anti‐TB) and healthy controls (HC); monocytes CD64 (mCD64) and lymphocytes CD64 (lymCD64) were used as the positive and negative controls, respectively. We then evaluated the clinical utility of the nCD64 index in the diagnosis of TB and finally evaluated serial changes in the nCD64 index during anti‐TB therapy in patients with active TB.

## 2 MATERIALS AND METHODS

### 2.1 Participants and diagnosis

**FIGURE 1:**
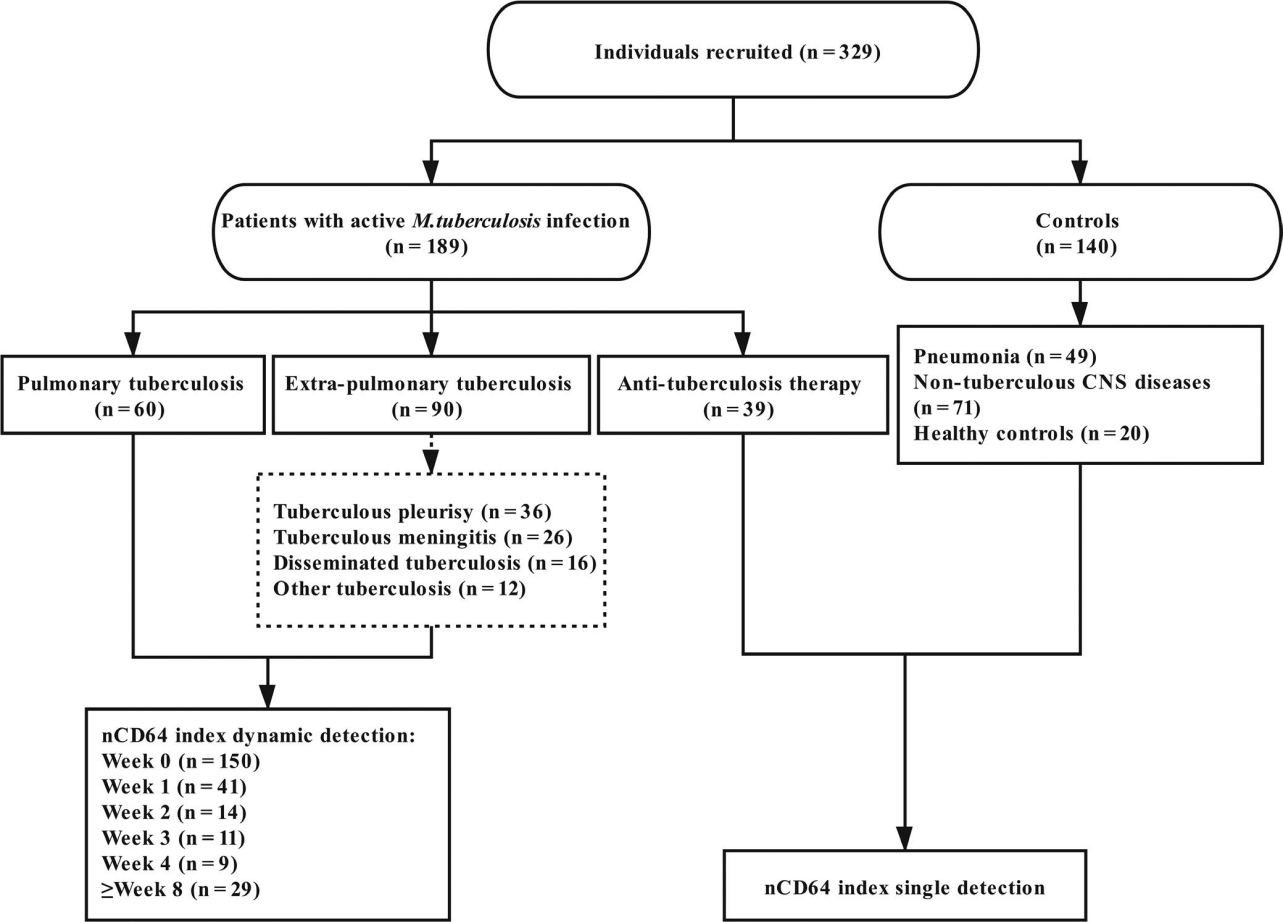
Study flow chart. Recruited individuals were divided into patients with active *M. tuberculosis*infection and controls. All TB patients were performed nCD64 index dynamic detection. *M. tuberculosis*, *Mycobacterium tuberculosis*; CNS, central nervous system

In this study, we recruited 329 individuals, comprising patients with active *M. tuberculosis* infection (n = 189) and controls (n = 140), from Fudan University Affiliated Huashan Hospital and Wuxi Fifth People's Hospital between October 2018 and November 2019. All individuals were free of human immunodeficiency virus (HIV) infection. Among the 189 patients with active TB, one‐third had pulmonary TB (PTB, n = 60); almost one‐half had extrapulmonary TB (ePTB, n = 90), which consisted of tuberculous pleurisy (TBP, n = 36), tuberculous meningitis (TBM, n = 26), disseminated TB (n = 16) and other ePTB (n = 12); and the remaining received anti‐TB therapy for more than one month at enrolment (n = 39). Patients with PTB and ePTB were followed up once a week from enrolment, and the flow chart is shown in Figure 1.

The diagnosis of TB was categorized as (a) confirmed diagnosis, with at least one of the following: acid‐fast bacilli (AFB) smear or *M. tuberculosis* culture positive in any specimen (sputum, pleural effusion, cerebral spinal fluid or blood), tuberculous granuloma suggested by histological examination of tissues, Xpert MTB/RIF positive, or next‐generation sequencing (NGS) detection of *M. tuberculosis*, and (b) clinical diagnosis, combination with clinical symptoms, radiological indicators and effective anti‐TB therapy when there is a lack of microbiology evidence.

The control group was categorized as (a) those with pneumonia (PN, n = 49), non‐tuberculous lung infections except for severe pneumonia, (b) those with non‐tuberculous CNS diseases, non‐tuberculous CNS infection (n = 64) and non‐infectious CNS diseases (n = 7) and (c) HC (n = 20), negative interferon‐γ release assay (IGRA) without evidence of active TB from the relatives of patients with TB and the volunteers of Huashan Hospital during the same period.

This study was approved by the Ethics committee of Huashan Hospital, Fudan University. Written informed consent was obtained from all participants.

### 2.2 Measurement and calculation of the nCD64 index

**FIGURE 2:**
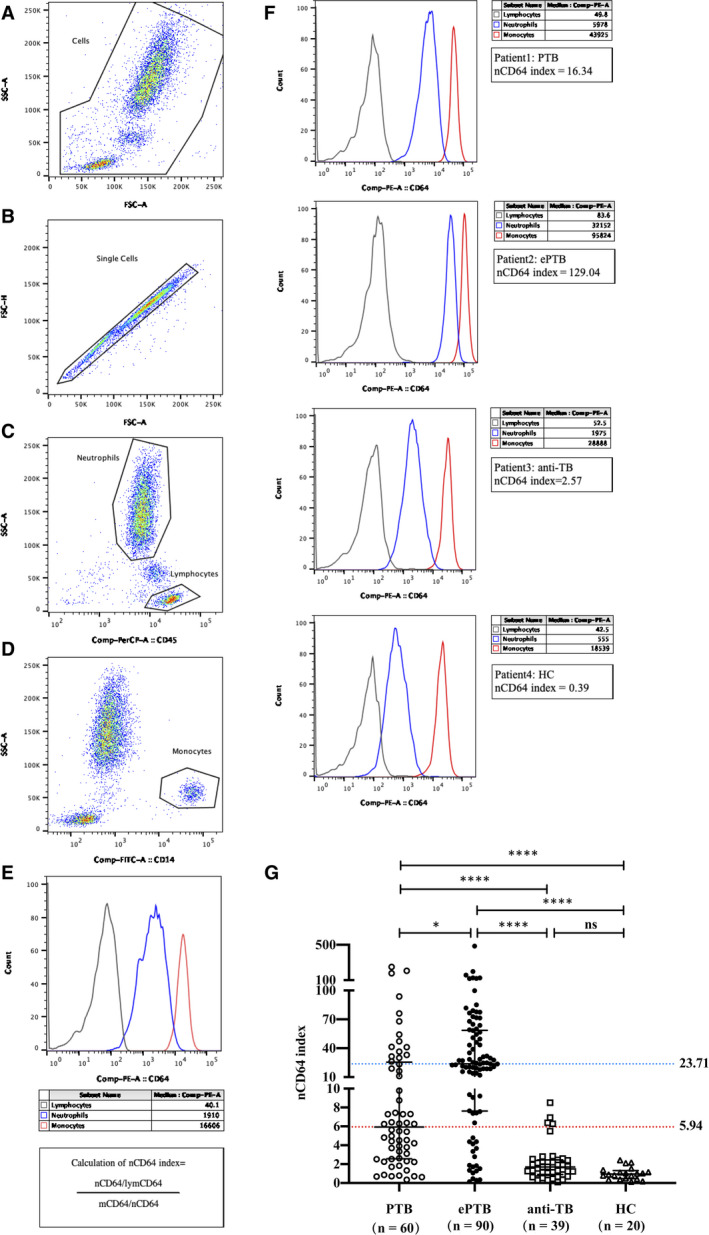
The differential expression of CD64 on leucocyte surface in patients with *M. tuberculosis*infection. (A) Cells were gated according to FSC and SSC plot. (B) Single cells were gated from (A) according to FSC‐A and FSC‐H plot. (C) Lymphocytes and neutrophils were gated from (B) according to pan‐leucocyte marker CD45 distribution. (D) Monocytes were gated from (B) according to CD14 distribution. (E) The histogram of CD64 expression (MFI) on neutrophils, lymphocytes and monocytes, and the formula of nCD64 index calculation. (F‐G) The differential expression of nCD64 index in patients with *M. tuberculosis*infection. Data were presented as medians and interquartile ranges. *P < .05, ****P < .0001. ePTB, extrapulmonary tuberculosis; FSC, forward scatter; HC, healthy control; MFI, median fluorescence intensity; ns, not significant; PTB, pulmonary tuberculosis; SSC, side scatter

Ethylenediaminetetraacetic acid (EDTA) anticoagulated peripheral blood samples (50 μL) were collected from the enrolled individuals, stored at room temperature (<24 hours) or 4°C (<48 hours) and then processed.^12^ The monoclonal antibodies of CD45‐PerCP, CD14‐FITC and CD45‐PE were purchased from BD Bioscience (San Jose, CA, USA). CD64 expression was measured by flow cytometry using FACSCanto Ⅱ (BD Bioscience, San Jose, CA, USA). The assay is simple and rapid, and it is based on whole‐blood cell‐surface staining. The pan‐leucocyte marker CD45 and monocyte marker CD14 were used to gate different WBC types according to their distribution in the forward side scatter (FSC) plot (Figure 2C,D). Furthermore, the expression of CD64 on WBC is presented as median fluorescence intensity (MFI), and the data were analysed using FlowJo™ Software 10 (BD Bioscience).

Given that CD64 is highly and stably expressed on the surface of monocytes and hardly expressed on lymphocytes, they can be used as the positive (mCD64 MFI) and negative (lymCD64 MFI) internal control, respectively (Figure S1). The CD64 index was calculated using the following formula (Figure 2A‐E): nCD64 index = (nCD64 MFI/lymCD64 MFI)/(mCD64 MFI/nCD64 MFI).

### 2.3 Stability of the nCD64 index in stored blood samples

For the convenience of clinical application, sometimes specimens have to be stored when they cannot be processed in time. Therefore, it is important that the nCD64 index value is stable in stored samples. To test this issue, we randomly selected 10 blood samples from patients with high or low nCD64 expression (nCD64 index < 1, n = 2; 1 ≤ nCD64 index < 10, n = 3; 10 ≤ nCD64 index < 50, n = 2; nCD64 index ≥ 50, n = 3), stored these samples at room temperature and determined the nCD64 index at 0 hour and after 24, 48, 72 and 96 hours.

### 2.4 T‐SPOT.TB assay

Peripheral blood mononuclear cells (PBMCs) were freshly isolated from anticoagulated blood samples by Ficoll density‐gradient centrifugation. The T‐SPOT.TB assay was performed according to the manufactures' (T‐SPOT.TB kit, Oxford Immune Ltd., Oxford, UK) instructions. The positive results were interpreted as previously described.^13^

### 2.5 Statistical analysis

Data were analysed using GraphPad Prism 8 (GraphPad, Inc., San Diego, CA, USA). Continuous variables were compared between independent groups using Mann–Whitney test (two groups) and Kruskal–Wallis test (multiple groups) followed by Dunn's post hoc test for multiple comparisons. Categorical variables were compared using the chi‐square test or Fisher's exact test, as appropriate. The follow‐up paired data were analysed using the paired Wilcoxon rank test. The receiver operating characteristics (ROC) curve analysis was performed to estimate the diagnostic performance of the nCD64 index and T‐SPOT.TB assay for the diagnosis of TB. The association between common inflammatory markers (WBC count, neutrophil percentage, ESR, CRP, PCT and ferritin) and nCD64 index was assessed with Spearman correlation. The results with a *P* value of <.05 were considered significant.

## 3 RESULTS

### 3.1 Individuals' characteristics

A total of 189 active patients with TB (PTB, ePTB and anti‐TB) were enrolled in this study; 41 out of the 60 (68.33%) and 33 out of the 90 (36.67%) patients in the PTB and ePTB groups were confirmed diagnosis cases, respectively. The anti‐TB therapy was administered for no more than two weeks in both PTB and ePTB groups at enrolment. The characteristics are summarized in Table 1. There were no significant differences in the clinical characteristics except for sex among the enrolled individuals; only 25% of the HC were men. Five out of the 189 (2.65%) patients died after enrolment.

**TABLE 1:** Clinical characteristics of enrolled individuals

Characteristics	PTB	ePTB	Anti‐TB^a^	HC	*P* value^b^
Number	60	90	39	20	/
Age, median (IQR), y	52 (31‐62)	45 (27‐65)	47 (28‐63)	28 (24‐52)	.1705
Male, n (%)	46 (76.67)	60 (66.67)	20 (51.28)	5 (25.00)	.0002
Female, n (%)	14 (23.33)	30 (33.33)	19 (48.72)	15 (75.00)	.0002
Confirmed diagnosis, n (%)	41 (68.33)	33 (36.67)	—	—	—
Microbiologically confirmed^c^	37 (61.67)	21 (23.33)	—	—	—
Xpert MTB/RIF positive	0 (0)	10 (11.11)	—	—	—
NGS positive	4 (6.67)	2 (2.22)	—	—	—
nCD64 index, median (IQR)	5.94 (2.56‐25.54)	23.71 (7.62‐58.59)	1.70 (0.90‐2.45)	0.96 (0.46‐1.32)	<.0001
TBP (n = 36) nCD64 index, median (IQR)	—	25.86 (9.25‐62.42)	—	—	—
TBM (n = 26) nCD64 index, median (IQR)	—	14.84 (3.94‐21.72)	—	—	—
Disseminated TB (n = 16) nCD64 index, median (IQR)	—	55.50 (27.54‐111.10)	—	—	—
Other (n = 12) nCD64 index, median (IQR)	—	20.65 (7.42‐75.25)	—	—	—

Note

Continuous variables presented as median (IQR); categorical variables presented as n (%).

Abbreviations: ePTB, extrapulmonary tuberculosis; HC, healthy control; IQR, interquartile range; NGS, next‐generation sequencing; PTB, pulmonary tuberculosis; TBM, tuberculous meningitis; TBP, tuberculous pleurisy.

^a^ Anti‐TB patients had been treated for more than one month at enrolment.

^b^ *P* values were determined with Kruskal‐Wallis test for continuous data and with chi‐square test for categorical data.

^c^ Microbiologically confirmed patients were diagnosed by culture or smear positive from any sample or confirmed histopathologically.

### 3.2 nCD64 index in patients with active *M. tuberculosis* infection

The nCD64 index was significantly higher in the active TB group (PTB median, 5.94; ePTB median, 23.71), than in the anti‐TB (median, 1.70) and HC (median, 0.96) groups (all *P* < .0001) (Table 1, Figure 2F,G). In particular, the nCD64 index in the ePTB group was almost four times higher than that in the PTB group (*P* = .0110). Furthermore, the nCD64 index was the highest in patients with disseminated TB (Table 1). There was no difference between the anti‐TB and HC groups.

**FIGURE 3:**
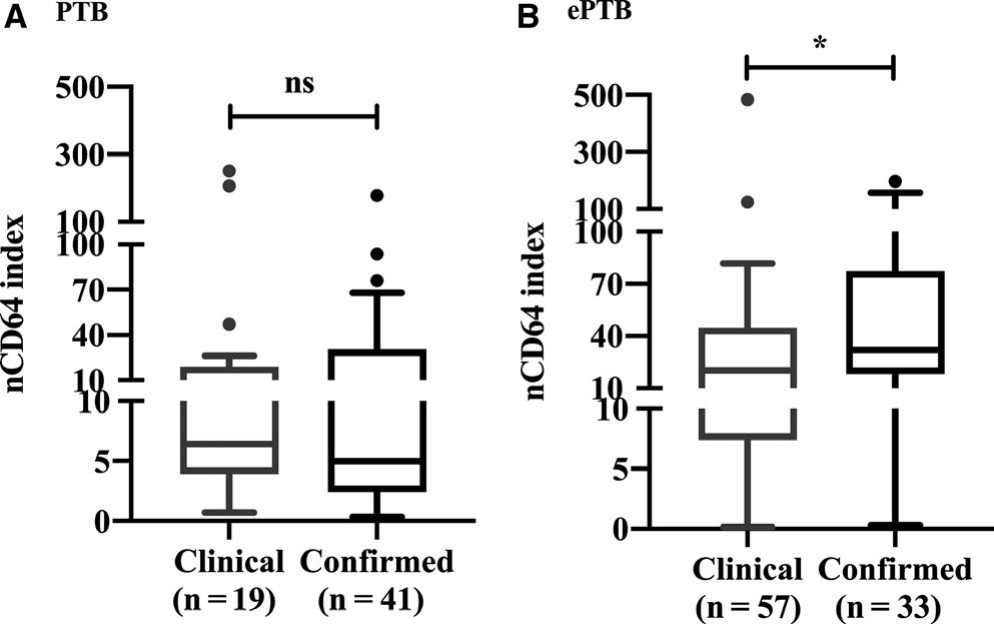
The difference of nCD64 index between clinical and confirmed diagnosis in patients with (A) PTB or (B) ePTB. The boxes show medians and interquartile ranges, whereas the whiskers indicate the 10th and 90th percentiles. *P < .05. ePTB, extrapulmonary tuberculosis; ns, not significant; PTB, pulmonary tuberculosis

Further analysis of the difference between clinical and confirmed diagnoses in patients with TB revealed that the nCD64 index was not significantly different in the PTB group (6.44 vs 4.98, *P* = .4657) (Figure 3A). However, in the ePTB group, the median of the nCD64 index was significantly higher in the confirmed diagnosis patients (31.90, n = 33) than in the clinical diagnosis patients (20.06, n = 57) (*P* = .0282; Figure 3B).

### 3.3 Utility of the nCD64 index in the diagnosis of PTB

**FIGURE 4:**
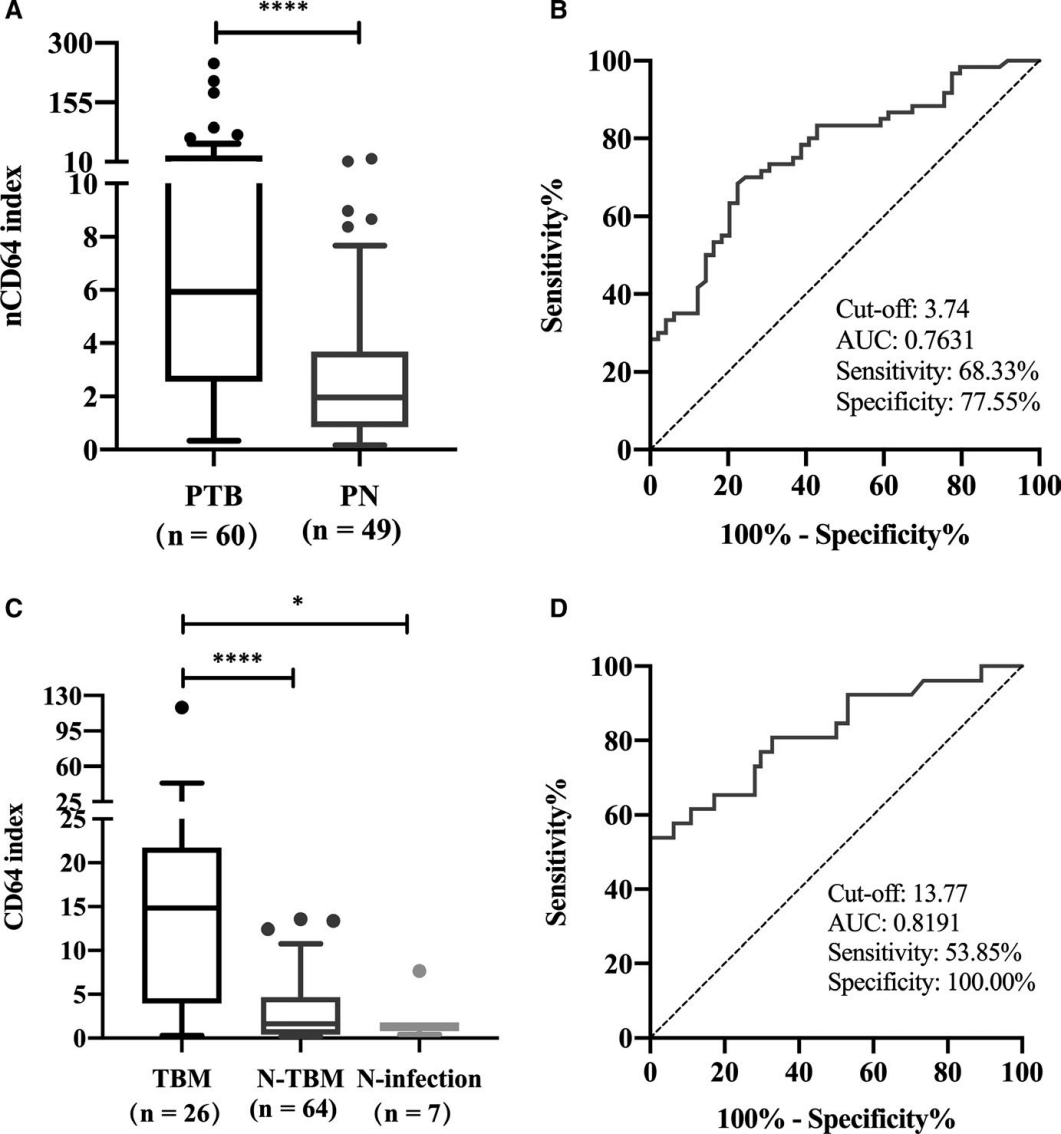
(A) nCD64 index expression in PTB and PN patients and (B) ROC curve analysis for the diagnosis of PTB. (C) nCD64 index expression among TBM, N‐TBM and N‐infection patients and (D) ROC curve analysis for distinguishing TBM from N‐TBM patients. The boxes show medians and interquartile ranges, whereas the whiskers indicate the 10th and 90th percentiles. *P < .05, ****P < .0001. AUC, area under the ROC curve; N‐infection, non‐infectious CNS diseases; N‐TBM, non‐tuberculous CNS infection; PN, pneumonia; PTB, pulmonary tuberculosis; ROC, receiver operating characteristic; TBM, tuberculous meningitis

To estimate the performance of the nCD64 index in the diagnosis of PTB, we enrolled 49 patients with non‐tuberculous PN, and among them, patients with severe PN were excluded (severe PN patients highly expressed nCD64, data were not shown) and collected their clinical characteristics (Table S1). As shown in Figure 4A, we first compared the median of the nCD64 index between PTB (5.94, 2.56‐25.54) and PN (1.96, 0.84‐3.69) patients and observed an approximately twofold increase in the nCD64 index in PTB patients (*P* < .0001). The ROC curve analysis yielded the cut‐off value of 3.74 for the nCD64 index with an area under the curve (AUC) of 0.7631 (95% CI, 0.6743‐0.8519, *P* < .0001), a sensitivity of 68.33% (95% CI, 55.77%–78.69%) and a specificity of 77.55% (95% CI, 64.12%–86.98%) (Figure 4B). Moreover, the T‐SPOT.TB assay and nCD64 index determination were simultaneously performed in 36 and the 37 cases in the PTB and PN groups, respectively. We used the cut‐off value of 3.74 for the diagnosis of PTB and compared the diagnostic accuracy of the nCD64 index with the T‐SPOT.TB assay in the differential diagnosis of PTB and PN patients (Table 2).

**TABLE 2:** Comparison of accuracy of T‐SPOT.TB and nCD64 index for the diagnosis of pulmonary tuberculosis

Assay^a^ (PTB = 36; PN = 37)	Values % (95% CI)				Ratio	
	Sensitivity	Specificity	PPV	NPV	Positive LR	Negative LR
T‐SPOT.TB	69.44 (53.14‐82.00)	64.86 (48.76‐78.17)	65.79 (49.89‐78.79)	68.57 (52.02‐81.45)	1.98	0.47
nCD64 index, cut‐off value of 3.74	63.89 (47.58‐77.52)	78.38 (62.80‐88.61)	74.19 (56.75‐86.30)	69.05 (53.97‐80.93)	2.96	0.46

Abbreviations: CI, confident interval; LR, likelihood ratio; NPV, negative predictive value; PN, pneumonia; PPV, positive predictive value; PTB, pulmonary tuberculosis.

^a^ The comparison of diagnostic accuracy was performed in 36 cases of PTB and 37 cases of PN patients, who were simultaneously tested for T‐SPOT.TB and nCD64 index

### 3.4 Utility of the nCD64 index in the diagnosis of TBM

To date, the differential diagnosis of CNS infection lacks specific biomarkers. We sought to understand whether the nCD64 index could be used to distinguish between TBM and non‐tuberculous CNS infection (N‐TBM). Therefore, we recruited 64 cases of N‐TBM patients and collected their clinical documents (Table S2), and the disease control group comprised patients with non‐infectious CNS diseases (N‐infection, n = 7). The nCD64 index of patients with TBM differed significantly from those with N‐TBM and N‐infection (14.84 vs 1.62 and 14.84 vs 1.34, *P* < .0001 and *P* = .0121, respectively) (Figure 4C, Table S2). Further analysis of the ROC was performed in TBM and N‐TBM patients, using the cut‐off value of 13.77; the AUC, sensitivity and specificity of the nCD64 index were 0.8191 (95% CI, 0.7142‐0.9240), 53.85% (95% CI, 35.46%–71.24%) and 100% (95% CI, 94.34%–100%), respectively (Figure 4D). We further used the cut‐off value of 13.77 for the diagnosis of TBM, compared the diagnostic accuracy of the nCD64 index with the T‐SPOT.TB assay, and found the specificity and PPV of the nCD64 index were superior to those of the T‐SPOT.TB assay (Table 3).

**TABLE 3:** Comparison of accuracy of T‐SPOT.TB and nCD64 index for the diagnosis of tuberculous meningitis

Assay^a^ (TBM = 26; N‐TBM = 57)	Values % (95% CI)				Ratio	
	Sensitivity	Specificity	PPV	NPV	Positive LR	Negative LR
T‐SPOT.TB	73.08 (53.92‐86.30)	77.19 (64.79‐86.16)	59.38 (42.26‐74.48)	86.27 (74.28‐93.19)	3.20	0.35
nCD64 index, cut‐off value of 13.77	53.85 (35.46‐71.24)	100.00 (93.69‐100.00)	100.00 (78.47‐100.00)	82.61 (72.02‐89.76)	8.53^b^	0.46

Abbreviations: CI, confident interval; LR, likelihood ratio; NPV, negative predictive value; N‐TBM, non‐tuberculous CNS infection; PPV, positive predictive value; TBM, tuberculous meningitis.

^a^ The comparison of diagnostic accuracy was performed in 26 cases of TBM and 57 cases of N‐TBM patients, who were simultaneously tested for T‐SPOT.TB and nCD64 index.

^b^ This value was calculated based on the lowest value of the specificity 95% CI (93.69%).

### 3.5 Correlation between the nCD64 index and clinical inflammatory indicators in patients with active *M. tuberculosis* infection

**FIGURE 5:**
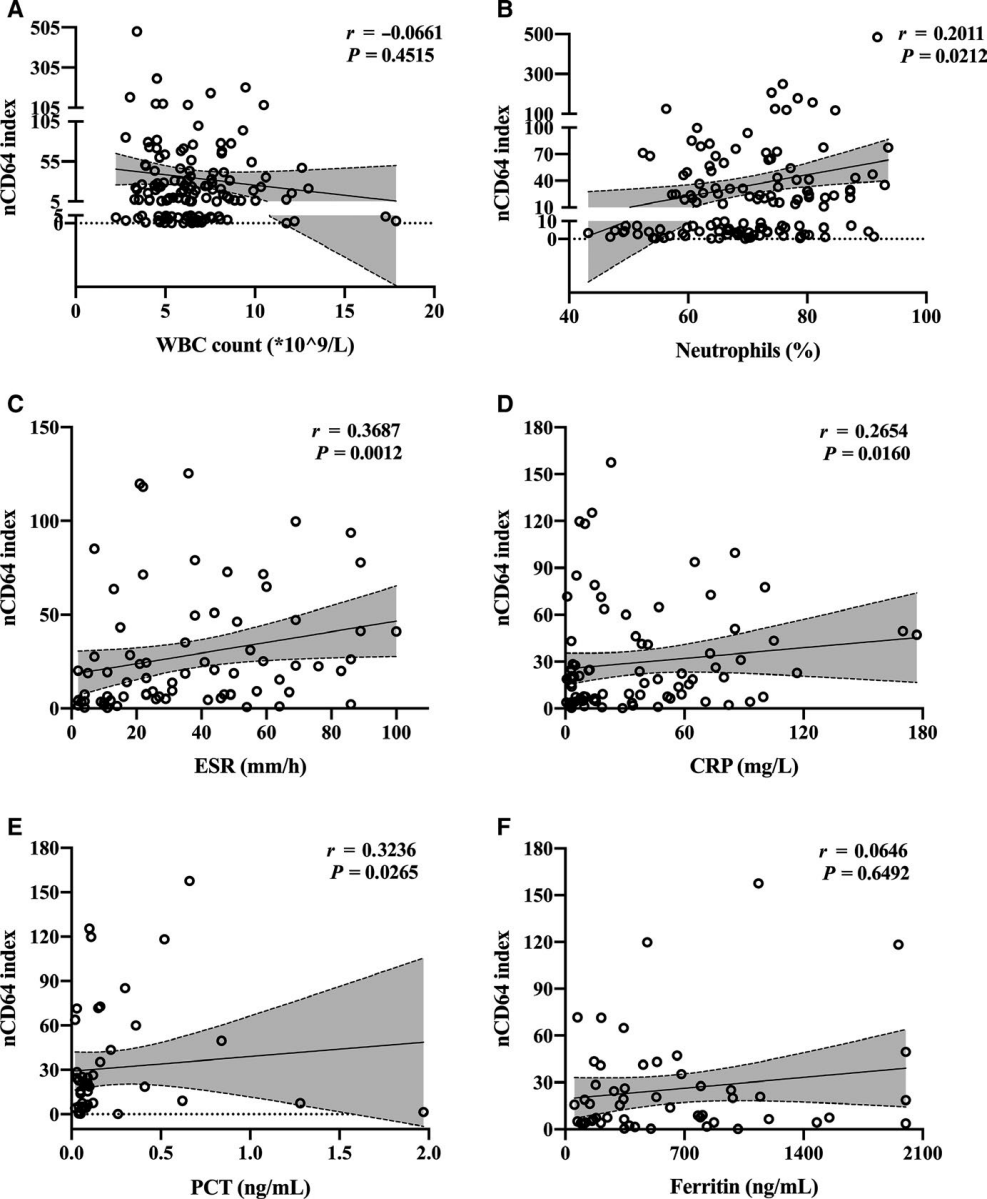
Correlations between nCD64 index and clinical inflammatory indicators in patients with TB including WBC count (A), neutrophils percentage (B), ESR (C), CRP (D), PCT (E) and ferritin (F). Spearman correlation analysis was used and linear curve fit (with 95% CI, grey interval) was used to illustrate trends of data distribution. CRP, C‐reactive protein; ESR, erythrocyte sedimentation rate; PCT, procalcitonin; TB, tuberculosis; WBC, white blood cell

We analysed whether the nCD64 index in patients with TB was correlated with clinical inflammatory indicators (WBC count, neutrophil percentage, ESR, CRP, PCT and ferritin). We observed that the nCD64 index value showed weak correlations with neutrophil percentage (*r* = 0.20, *P* = .0212) (Figure 5B), ESR (*r* = 0.37, *P* = .0012) (Figure 5C), CRP (*r* = 0.27, *P* = .0160) (Figure 5D) and PCT (*r* = 0.32, *P* = .0265) (Figure 5E). However, there were no correlations between the nCD64 index and WBC count and ferritin (Figure 5A,F).

### 3.6 Dynamic determinations of the nCD64 index and prognosis in patients with active *M. tuberculosis* infection

**FIGURE 6:**
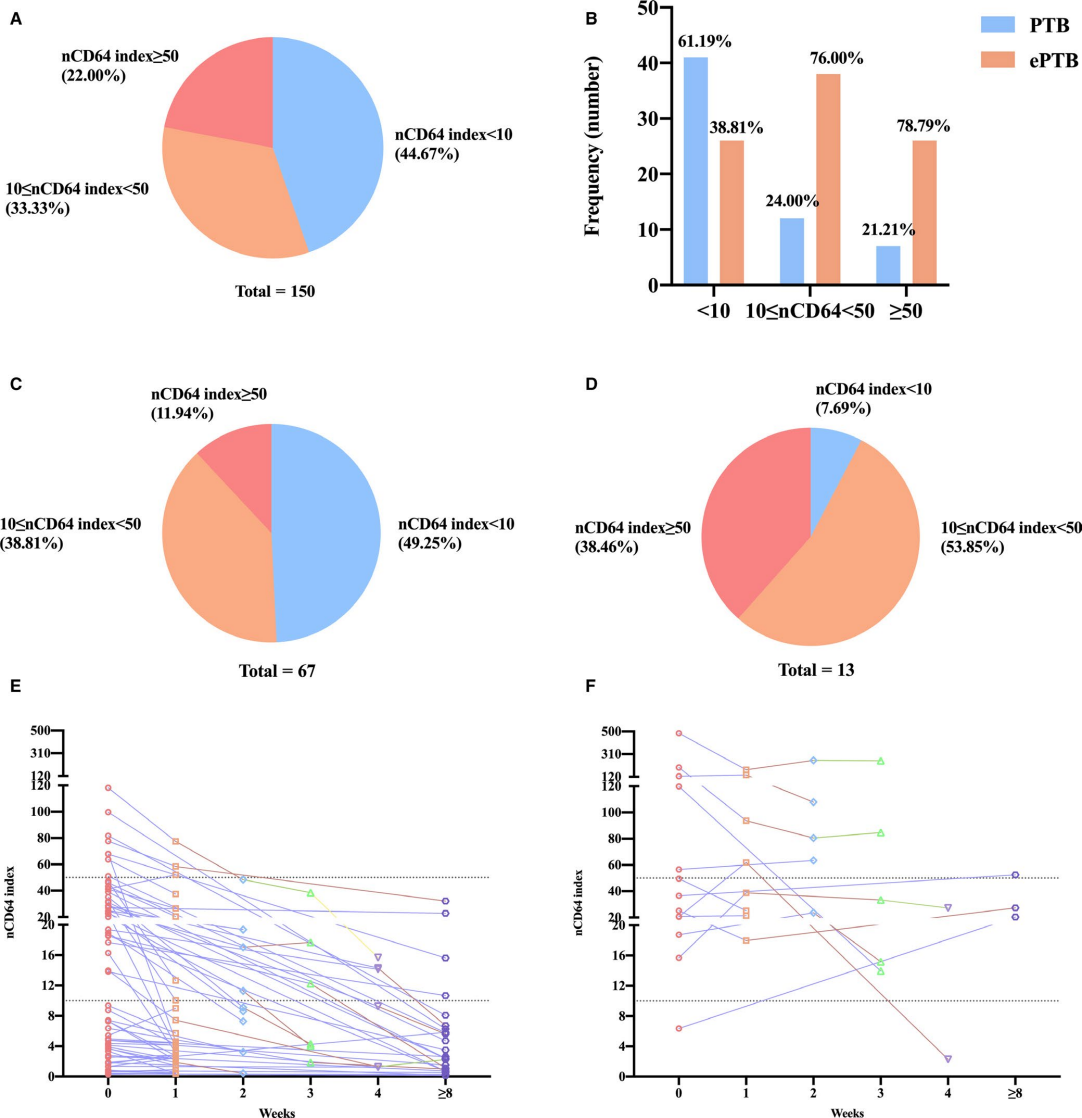
(A) Pie chart showed the frequency of active TB patients who had low (<10), moderate (≤nCD64 index < 50) and high (≥50) nCD64 index value. (B) The frequency distribution of PTB and ePTB patients among different levels of nCD64 index group. Pie chart showed the frequency of improvement (C) and poor improvement (D) patients who had low (<10), moderate (≤nCD64 index < 50) and high (≥50) nCD64 index value. Dynamic determinations of nCD64 index in active TB patients from improvement (E) and poor improvement group (F), the upper and lower dotted line represented nCD64 index = 50 and 10, respectively

We further divided all the patients into three groups based on the baseline nCD64 index values, as shown in Figure 6A,B. Among all TB patients, 44.67% had the nCD64 index of <10, in which 61.19% and 38.81% were from PTB and ePTB group, respectively. However, the patients with PTB accounted for less than one‐fourth of those in the 10 ≤ nCD64 index < 50 (24.00%) and nCD64 index ≥ 50 groups (21.21%) (Figure 6B). Furthermore, as we expected, the percentage of ePTB patients in the above two groups with high nCD64 index exceeded 75% (Figure 6B). This suggested that high nCD64 index values might be associated with the dissemination of TB.

Among the 189 patients with active *M. tuberculosis* infection at enrolment, 5 (2.65%) died during the follow‐up, and all were from the ePTB group; three patients died of multiple organ failure (MOF), one died of upper gastrointestinal haemorrhage, and one died of cerebral haemorrhage.

We had data of at least one follow‐up of 80 (53.33%) patients with active TB. According to the efficiency of treatment, the 80 patients were divided into improvement (n = 67) and poor improvement (n = 13, including 4 deaths) groups. Notably, 97.06%, 78.79% and 61.54% of the nCD64 index < 10, 10 ≤ nCD64 index < 50 and nCD64 index ≥ 50 patients, respectively, were included in the improvement group (*P* = .0077); moreover, the proportion of patients with high nCD64 index values significantly differed between the improvement and poor improvement groups (Figure 6C,D). The nCD64 index value started to significantly decrease at 1 week of anti‐TB treatment in the improvement group, reaching up to 10, and it evidently decreased to below 10 at 8 weeks of treatment (*P* < .0001, Figure 6E). However, in the poor improvement group, there were no significant differences in the nCD64 index at the baseline and 1, 2 and 3 week(s) (all *P* > .05, Figure 6F).

### 3.7 Qualification of stability of the nCD64 index in stored blood samples

**FIGURE 7:**
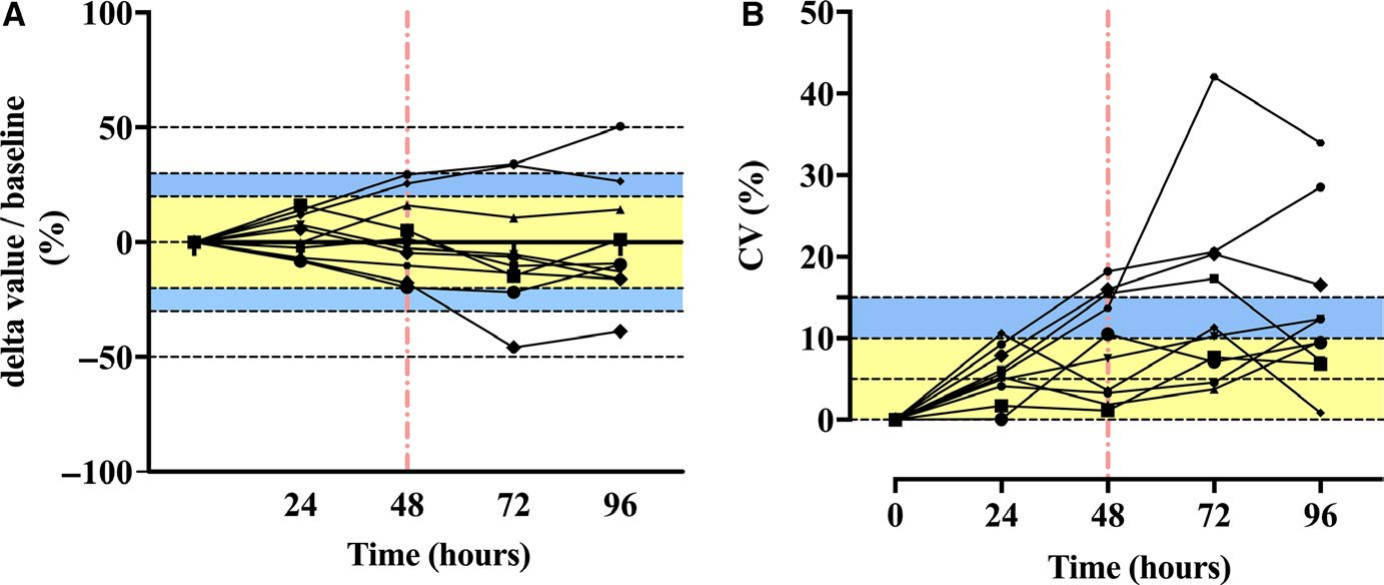
Stability qualification of nCD64 index in stored blood samples at different time‐points. (A) The percentage of nCD64 index delta value at different time‐points accounting for baseline, delta value = nCD64 index at different time‐points‐ nCD64 index at 0 h; yellow and blue area represents ±20% and ±30%, respectively. (B) The CV value at different time‐points; yellow and blue area represents within 10% and 15%, respectively. The red line represents acceptable results within 48 h stored blood samples at room temperature. CV, coefficient of variance

As shown in Figure 7A, we evaluated the stability of the nCD64 index based on the delta value and observed that the absolute delta value percentage was within 20% and the coefficient of variation (CV) was less than 10% when the blood samples were stored for <24 hours at room temperature (Figure 7B). Thus, we recommend that the blood samples should not be stored for more than 48 hours at room temperature (delta value, 30%; CV, 15%), and they can be stored at 4°C for a short period when necessary.

## 4 DISCUSSION

In this study, we investigated the differential expression of nCD64 in patients with PTB and ePTB and observed that patients with *M. tuberculosis* infection highly expressed nCD64 when compared with the anti‐TB patients and HC. Furthermore, the nCD64 index was considerably higher in patients with ePTB than in those with PTB, and more importantly, it was elevated up to 10‐fold in patients with disseminated TB, indicating that there might be an association between the nCD64 index and TB dissemination. Our results showed there was a significant difference in sex among these groups, which might be due to the higher incidence of tuberculosis in men than in women, but it is reported that sex had almost no effect on the number of granulocytes, monocytes and CD64 expression^14^; therefore, we thought the differences in sex composition would not affect the nCD64 index. Additionally, the nCD64 index value varied with treatment outcome, and an apparent decrease in the nCD64 index might suggest that the treatment was effective. To verify whether the type of diagnosis influences the nCD64 index, we compared the nCD64 index between confirmed and clinical diagnosis patients with active TB (PTB and ePTB). Interestingly, our results showed a higher nCD64 index in confirmed diagnosis than in clinical diagnosis ePTB patients, but there was no significant difference between confirmed and clinical diagnosis patients in the PTB group. This can be attributed to the following factors. First, clinical or empirical diagnosis of ePTB (63.33%) is more common than PTB (31.67%) in a clinical setting, because the culture of lesion samples depends on invasive operation. Second, confirmed diagnosis patients with ePTB based on culture positivity might have a higher burden of *M. tuberculosis* than clinical diagnosis patients.

It has been reported that *FCGRIA* (a *CD64* gene) could be used as a candidate biomarker to diagnose TB in several gene expression signatures studies, irrespective of peripheral blood monocular cells (PBMCs) or whole‐blood samples.^15^, ^16^, ^17^, ^18^ A previous study of 523 participants across four African countries demonstrated that *FCGRIA* was the only marker expressed at a higher level in active TB patients than in latent TB (LTBI) patients, regardless of the HIV status or ethnicity, with the AUC of 0.75 and 0.87 in HIV‐positive and HIV‐negative participants, respectively. Therefore, it can be used to distinguish active TB from LTBI.^16^ Additionally, the protein expression of CD64 on monocytes increased compared with that in PBMC samples in TB patients.^15^ In contrast, another whole‐blood transcriptomic study showed a significant decrease in *CD64* expression in patients with PTB when compared with patients with other pulmonary diseases.^17^ In this study, we calculated the nCD64 index value considering the expression of CD64 on neutrophils, monocytes and lymphocytes, which better met the previously described property of CD64.

Importantly, to differentiate tuberculous from non‐tuberculous lung infections, we used the cut‐off value of 3.74 for the nCD64 index to diagnose PTB patients; the sensitivity (63.89%) was lower and specificity (78.38%) was higher than that of the T‐SPOT.TB assay (69.44% and 64.86%, respectively). Similarly, the nCD64 index was recently assessed for the differential diagnosis of active TB and LTBI using the Leuko64™ kit, with a sensitivity of 80.00% and specificity of 76.92%, respectively.^19^ Previous studies rarely assessed the diagnostic value of the nCD64 index for the differential diagnosis of CNS infection when the lesions were in the meninges. Notably, in this study, we recruited 26 cases of TBM without dissemination and demonstrated that patients with TBM can be distinguished from those with CNS infection by the higher nCD64 index with the cut‐off value of 13.77; the PPV was 100%, which was superior to that of the T‐SPOT.TB assay (PPV, 59.38%). Although empirical diagnoses are often used by clinicians, the diagnostic gold standard of CNS infection is cerebral spinal fluid (CSF)‐dependent culture, which inevitably requires an invasive operation. The nCD64 index detection was found to be superior to the T‐SPOT.TB study. Moreover, it can be completed within 1 hour after sample collection and has a greater PPV for the diagnosis of TBM. This unexpected finding provides a clue for the diagnosis of TB; thus, the method can avoid some unnecessary empiric therapeutic decisions by clinicians.

As reported earlier, the nCD64 index has been shown to be associated with the severity of inflammatory responses in sepsis and inflammatory disorders.^20^, ^21^ Our results indicated weak correlations between common inflammatory indicators (ESR, PCT, CRP and neutrophil percentage) and the nCD64 index, and this might be explained by chronic *M. tuberculosis* infection, which is different from acute bacterial infection or inflammatory cascade responses. We observed the nCD64 index was less than 10 in almost one‐half (49.25%) of patients with improvement and in only 7.69% of patients with poor improvement. On the contrary, the percentage of nCD64 index ≥ 50 in the poor improvement group (38.46%) was three times higher than that in the improvement group (11.94%). Moreover, with the improvement in patient condition, the nCD64 index started to decline in the first week of anti‐TB therapy and significantly decreased at 4 weeks after treatment. However, in patients with poor improvement, including deaths, there was no apparent difference between the baseline and follow‐up. Therefore, the dynamic detection of nCD64 index could reflect therapeutic efficacy and even serve as a prognostic marker.

Currently, the role of CD64 in *M. tuberculosis* infection is still unclear. CD64, is strongly induced by proinflammatory cytokines such as G‐CSF and IFN‐γ, and expressed on the surface of activated neutrophils, which play important roles in phagocytosing IgG‐opsonized bacteria,^22^ inducing reactive oxygen species (ROS) production, triggering antibody‐dependent cytotoxicity (ADCC)^22^, ^23^ and clearing the immune complex. It also facilitates to control *M. tuberculosis* by stimulating the respiratory burst in intracellular mononuclear phagocytes. Moreover, Fcγ receptor can affect cytokine production and susceptibility during *M. tuberculosis* infection.^24^ Previously, it has been reported that the increase in CD64 was associated with increased phagocytosis capacity and inversely correlated with TNF‐α secretion in inflammatory pathology.^14^ Consequently, the up‐regulation of CD64 likely indicates the immunopathogenesis in patients with *M. tuberculosis* infection.

Finally, considering the convenience of clinical application, we measured the stability of the nCD64 index in blood samples stored at room temperature for 0, 24, 48 and 72 hours even to 96 hours. Our results indicated the stability was the best within 24 h in stored blood samples at room temperature, with less than 10% of CV, and worse when stored for more than 48 hours. Furthermore, the stability can prolong to 72 hours when blood samples were stored at 4°C^12^, ^25^

Our study had some limitations. First, the number of participants recruited in this study was relatively small, and larger number of individuals should be evaluated in the future. Second, we aimed to distinguish patients with PTB from those with non‐tuberculous lung infection; thus, we did not estimate the utility of the nCD64 index for the differential diagnosis of PTB and LTBI. Third, we did not explore the CD64‐associated mechanism involved in *M. tuberculosis* infection, and it should be explored in the future. Despite these limitations, our study presented robust data indicating that the nCD64 index could serve as a potential biomarker for the clinical diagnosis and prognosis of patients with TB.

In conclusion, our study demonstrated that the nCD64 index can be a potential biomarker to distinguish between PTB and PN patients and to identify patients with TBM from those with CNS infection, with the higher specificity and PPV, which were superior to those of the T‐SPOT.TB assay. Moreover, this fast, non‐invasive and stable method is convenient for clinical application. More importantly, the nCD64 index can reflect the responses to anti‐TB treatment and serve as a therapeutic monitor.

## CONFLICT OF INTEREST

All authors declare that there is no conflict of interest.

## AUTHOR CONTRIBUTION

**Qianqian Liu:** Data curation (equal); Formal analysis (lead); Investigation (equal); Writing‐original draft (lead). **Yan Gao:** Investigation (equal); Project administration (equal). **Qinfang Ou:** Resources (lead). **Yuzhen Xu:** Data curation (supporting); Investigation (supporting). **Zhe Zhou:** Data curation (supporting); Investigation (supporting). **Ting Li:** Methodology (equal). **Yi Lu:** Formal analysis (supporting); Methodology (supporting). **Feng Sun:** Resources (supporting). **Xian Zhou:** Resources (supporting). **Yang Li:** Resources (supporting). **Lingyun Shao:** Conceptualization (equal); Funding acquisition (equal); Writing‐review & editing (equal). **Wen‐Hong Zhang:** Conceptualization (equal); Funding acquisition (equal); Supervision (lead).

## Supporting information

Appendix S1

## Data Availability

The data are available from the corresponding author upon reasonable request.

## References

[1] Sack U . CD64 expression by neutrophil granulocytes. Cytometry B Clin Cytom. 2017;92:189‐191. 25522066 10.1002/cyto.b.21216

[2] Wang Y , Jonsson F . Expression, role, and regulation of neutrophil Fcgamma receptors. Front Immunol. 2019;10:1958. 31507592 10.3389/fimmu.2019.01958 PMC6718464

[3] Mortaz E , Alipoor SD , Adcock IM , et al. Update on neutrophil function in severe inflammation. Front Immunol. 2018;9:2171. 30356867 10.3389/fimmu.2018.02171 PMC6190891

[4] Aleman M , de la Barrera SS , Schierloh PL , et al. In tuberculous pleural effusions, activated neutrophils undergo apoptosis and acquire a dendritic cell‐like phenotype. J Infect Dis. 2005;192:399‐409. 15995953 10.1086/431680

[5] Wang X , Li ZY , Zeng L , et al. Neutrophil CD64 expression as a diagnostic marker for sepsis in adult patients: a meta‐analysis. Critical care (London, England). 2015;19:245. 10.1186/s13054-015-0972-z PMC4490738 26059345

[6] Dimoula A , Pradier O , Kassengera Z , et al. Serial determinations of neutrophil CD64 expression for the diagnosis and monitoring of sepsis in critically ill patients. Clin Infect Dis. 2014;58:820‐829. 24363321 10.1093/cid/cit936

[7] Gibot S , Bene MC , Noel R , et al. Combination biomarkers to diagnose sepsis in the critically ill patient. Am J Respir Crit Care Med. 2012;186:65‐71. 22538802 10.1164/rccm.201201-0037OC

[8] Jamsa J , Ala‐Kokko T , Huotari V , et al. Neutrophil CD64, C‐reactive protein, and procalcitonin in the identification of sepsis in the ICU – Post‐test probabilities. J Crit Care. 2018;43:139‐142. 28898742 10.1016/j.jcrc.2017.08.038

[9] Nuutila J , Hohenthal U , Oksi J , et al. A single‐tube two‐color flow cytometric method for distinguishing between febrile bacterial and viral infections. J Microbiol Methods. 2018;152:61‐68. 30063957 10.1016/j.mimet.2018.07.015

[10] Gros A , Roussel M , Sauvadet E , et al. The sensitivity of neutrophil CD64 expression as a biomarker of bacterial infection is low in critically ill patients. Intensive Care Med. 2012;38:445‐452. 22310872 10.1007/s00134-012-2483-6

[11] Cid J , Aguinaco R , Sanchez R , et al. Neutrophil CD64 expression as marker of bacterial infection: a systematic review and meta‐analysis. J Infect. 2010;60:313‐319. 20206205 10.1016/j.jinf.2010.02.013

[12] Davis BH . Improved diagnostic approaches to infection/sepsis detection. Expert Rev Mol Diagn. 2005;5:193‐207. 15833049 10.1586/14737159.5.2.193

[13] Shao L , Zhang W , Zhang S , et al. Potent immune responses of Ag‐specific Vgamma2Vdelta2+ T cells and CD8+ T cells associated with latent stage of *Mycobacterium tuberculosis* coinfection in HIV‐1‐infected humans. AIDS. 2008;22:2241‐2250. 18981763 10.1097/QAD.0b013e3283117f18 PMC2743094

[14] Puissant‐Lubrano B , Apoil PA , Guedj K , et al. Distinct effect of age, sex, and CMV seropositivity on dendritic cells and monocytes in human blood. Immunol Cell Biol. 2018;96:114‐120. 29359459 10.1111/imcb.1004

[15] Jacobsen M , Repsilber D , Gutschmidt A , et al. Candidate biomarkers for discrimination between infection and disease caused by *Mycobacterium tuberculosis* . J Mol Med (Berl). 2007;85:613‐621. 17318616 10.1007/s00109-007-0157-6

[16] Sutherland JS , Loxton AG , Haks MC , et al. Differential gene expression of activating Fcgamma receptor classifies active tuberculosis regardless of human immunodeficiency virus status or ethnicity. Clin Microbiol Infect. 2014;20:O230‐O238. 24205913 10.1111/1469-0691.12383

[17] Laux da Costa L , Delcroix M , Dalla Costa ER , et al. A real‐time PCR signature to discriminate between tuberculosis and other pulmonary diseases. Tuberculosis. 2015;95:421‐425. 26025597 10.1016/j.tube.2015.04.008 PMC4475479

[18] Singhania A , Wilkinson RJ , Rodrigue M , et al. The value of transcriptomics in advancing knowledge of the immune response and diagnosis in tuberculosis. Nat Immunol. 2018;19:1159‐1168. 30333612 10.1038/s41590-018-0225-9 PMC6554194

[19] Correa RDS , Rodrigues LS , Pereira LHL , et al. Neutrophil CD64 expression levels in IGRA‐positive individuals distinguish latent tuberculosis from active disease. Mem Inst Oswaldo Cruz. 2019;114:e180579. 30970080 10.1590/0074-02760180579 PMC6454854

[20] ten Oever J , Netea MG , Kullberg BJ . Utility of immune response‐derived biomarkers in the differential diagnosis of inflammatory disorders. J Infect. 2016;72:1‐18. 10.1016/j.jinf.2015.09.007 26429736

[21] Yeh CF , Wu CC , Liu SH , et al. Comparison of the accuracy of neutrophil CD64, procalcitonin, and C‐reactive protein for sepsis identification: a systematic review and meta‐analysis. Ann Intensive Care. 2019;9:5. 30623257 10.1186/s13613-018-0479-2 PMC6325056

[22] Schiff DE , Rae J , Martin TR , et al. Increased phagocyte Fc gammaRI expression and improved Fc gamma‐receptor‐mediated phagocytosis after in vivo recombinant human interferon‐gamma treatment of normal human subjects. Blood. 1997;90:3187‐3194. 9376602

[23] Repp R , Valerius T , Sendler A , et al. Neutrophils express the high affinity receptor for IgG (Fc gamma RI, CD64) after in vivo application of recombinant human granulocyte colony‐stimulating factor. Blood. 1991;78:885‐889. 1714327

[24] Maglione PJ , Xu J , Casadevall A , et al. Fc gamma receptors regulate immune activation and susceptibility during *Mycobacterium tuberculosis* infection. J Immunol. 2008;180:3329‐3338. 18292558 10.4049/jimmunol.180.5.3329

[25] Tillinger W , Jilch R , Jilma B , et al. Expression of the high‐affinity IgG receptor FcRI (CD64) in patients with inflammatory bowel disease: a new biomarker for gastroenterologic diagnostics. Am J Gastroenterol. 2009;104:102‐109. 19098857 10.1038/ajg.2008.6

