# Supplementary material for: Differential expression of CD64 in patients with Mycobacterium tuberculosis infection: A potential biomarker for clinical diagnosis and prognosis
Source: J Cell Mol Med. 2020 Nov 8;24(23):13961–72. doi: 10.1111/jcmm.16004 (PMC7753880; doi:10.1111/jcmm.16004)
Supplement: Supplementary file 1 — Appendix S1 [file JCMM-24-13961-s001.docx]

**Supplementary materials**


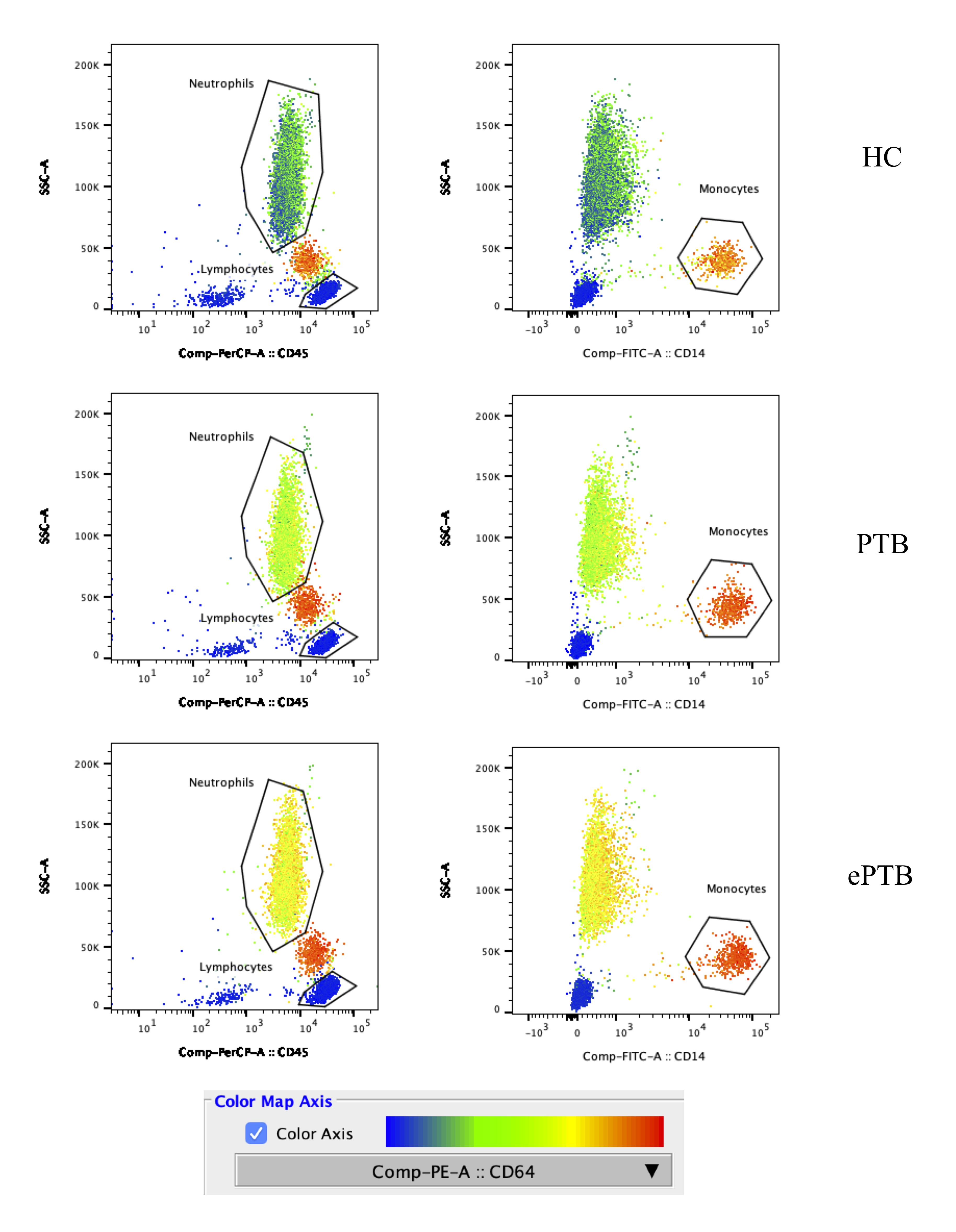


**Figure S1.** The rationale of nCD64 index for the utility in the diagnosis of TB. Representative dot plots of different WBC subpopulations (neutrophil, monocyte and lymphocyte) based on the expression levels of CD64. The pan-leukocyte marker CD45 and SSC were used to gate neutrophils and lymphocytes (left panel), and CD14 expression and SSC were used to define monocytes (right panel). The expression levels of CD64 on different cell types were presented based on the color map axis. CD64 hardly expressed on the surface of lymphocytes (blue) regardless of *M. tuberculosis* infection status, thus lymCD64 was served as the negative internal control; moreover, CD64 highly expressed on the surface of monocytes (red) regardless of *M. tuberculosis* infection status, thus mCD64 was served as the positive internal control; more importantly, CD64 levels on the surface of neutrophils varied with *M. tuberculosis* infection status, gradually elevated from HC to ePTB (from green to yellow) (left panel). HC, healthy control; PTB, pulmonary tuberculosis; ePTB, extrapulmonary tuberculosis; WBC, white blood cell; SSC, side scatter.

**Table S1. Clinical characteristics of pulmonary tuberculosis and pneumonia patients**

| Characteristics | PTB | PN | *P* value^a^ |
| --- | --- | --- | --- |
| Number | 60 | 49 | - |
| Age, median (IQR), y | 52 (31-62) | 62 (52-76) | **<0.0001** |
| Male, n (%) | 46 (76.67) | 35 (71.43) | 0.6600 |
| Female, n (%) | 14 (23.33) | 14 (28.57) | 0.6600 |
| T-SPOT.TB assay | 36 (60.00) | 37 (75.51) | 0.1038 |
| nCD64 index, median (IQR) | 5.94 (2.56-25.54) | 1.96 (0.84-3.69) | **<0.0001** |

Abbreviations: PTB, pulmonary tuberculosis; PN, pneumonia; IQR, interquartile range; TB, tuberculosis.

^a^*P* values were determined with Mann-Whitney test for continuous data and with Fisher’s exact test for categorical data.

**Table S2. Clinical characteristics of central nervous system infection and non-infection patients**

| Characteristics | TBM | N-TBM^a^ | N-infection^b^ | *P* value^c^ |
| --- | --- | --- | --- | --- |
| Number | 26 | 64 | 7 | - |
| Age, median (IQR), y | 47 (29-68) | 45 (30-59) | 48 (31-69) | 0.2272 |
| Male, n (%) | 16 (61.54) | 41 (64.06) | 5 (71.43) | 0.8145 |
| Female, n (%) | 10 (38.46) | 23 (35.94) | 2 (28.57) | 0.8145 |
| T-SPOT.TB assay, n (%) | 26 (100.00) | 57 (89.06) | 6 (85.71) | 0.1030 |
| nCD64 index, median (IQR) | 14.84 (3.94-21.72) | 1.62 (0.43-4.66) | 1.34 (0.76-1.77) | **<0.0001** |
| CSF examination, median (IQR) | | | | |
| CSF/blood glucose ratio | 0.29 (0.23-0.44) | 0.43 (0.27-0.54) | 0.37 (0.20-0.62) | **0.0396** |
| Chloride, mmol/L | 109 (103-117) | 117 (113-121) | 121 (116-128) | **0.0008** |
| Protein, mg/L | 3455 (1488-5740) | 1092 (634-2407) | 1347 (670-4728) | **0.0001** |
| WBC count, *10^6/L | 132 (53-355) | 66 (26-185) | 51 (1-147) | 0.0797 |

Abbreviations: TBM, tuberculous meningitis; IQR, interquartile range; TB, tuberculosis; CSF, cerebrospinal fluid; WBC, white blood cell; CNS, central nervous system.

^a^N-TBM group included bacterial (n=27), viral (n=28), cryptococcal (n=5) and other (n=4) CNS infection.

^b^N-infection group included CNS tumor (n=3) and other non-infectious CNS diseases (n=4).

^c^*P* values were determined between TBM and N-TBM group with Mann-Whitney test for continuous data and with Fisher’s exact test for categorical data.
